# An Alternative to Mapping a Word onto a Concept in Language Acquisition: Pragmatic Frames

**DOI:** 10.3389/fpsyg.2016.00470

**Published:** 2016-04-19

**Authors:** Katharina J. Rohlfing, Britta Wrede, Anna-Lisa Vollmer, Pierre-Yves Oudeyer

**Affiliations:** ^1^Cognitive Interaction Technology, Bielefeld UniversityBielefeld, Germany; ^2^Faculty of Arts and Humanities, Paderborn UniversityPaderborn, Germany; ^3^INRIABordeaux, France

**Keywords:** language acquisition, pragmatics, infants’ social learning, frames, learning and memory, developmental robotics

## Abstract

The classic mapping metaphor posits that children learn a word by mapping it onto a concept of an object or event. However, we believe that a mapping metaphor cannot account for word learning, because even though children focus attention on objects, they do not necessarily remember the connection between the word and the referent unless it is framed pragmatically, that is, within a task. Our theoretical paper proposes an alternative mechanism for word learning. Our main premise is that word learning occurs as children accomplish a goal in cooperation with a partner. We follow Bruner’s (1983) idea and further specify pragmatic frames as the learning units that drive language acquisition and cognitive development. These units consist of a sequence of actions and verbal behaviors that are co-constructed with a partner to achieve a joint goal. We elaborate on this alternative, offer some initial parametrizations of the concept, and embed it in current language learning approaches.

## Introduction

The direct mapping of words onto concepts has often been considered to be at the core of the language acquisition mechanism. Indeed, it has been suggested that “in order to successfully acquire a new word, young children must learn the correct associations between labels and their referents” (Wojcik, 2013, p. 1). Children must first “attend to and encode information about the referent,” and they have to learn “how the sounds in their language map onto objects, actions, and other properties of the world” (Wojcik, 2013, p. 1). Studies investigating this mechanism assume that once children are able to perceive a referent and to hear the word given to it, they will (1) link the word with the referent and (2) remember it as a mapping. Then when they see the referent in another situation, they will be able to recall the word; vice versa, when they hear the word, they will be able to recall a memory of the object.

However, recent studies on language success in children from low-income families indicate that the ability to refer a word to a referent might be only the last link in a long chain that first starts with establishing more fundamental communicative skills that have been called “communication foundations” (Bruner, 1983; Stephens and Matthews, 2014; Hirsh-Pasek et al., 2015, p. 2). Nonetheless, many questions regarding the nature of these communication foundations remain open. We believe that improving our understanding of these foundations will advance an alternative explanation of how children acquire language in a social interaction.

Although there are certainly plenty of situations in children’s everyday lives in which a novel word is taught to them by depicting and labeling a novel object, we suggest that this is not how word learning starts. Children learn the word “binky” not because it is a novel word introduced to them before its use, but because it is a word for an object that is used in the context of such activities as soothing that involve not only the child but also other persons. Importantly, the main purpose of these activities is not to acquire a new word but to achieve a joint action goal (Lock, 1978; Bruner, 1983). Hence, children will pick up this word because it is being used for a particular purpose that is relevant to them, and because it is uttered hundreds of times to express activities in which it is involved. Consequently, action and language are interwoven right from the start (Lock, 1978). Moreover, this interaction is organized systematically (e.g., Nomikou and Rohlfing, 2011) and constrained by culture (Shotter and Newson, 1982; Bruner, 1983; Nelson, 2007).

**FIGURE 1:**
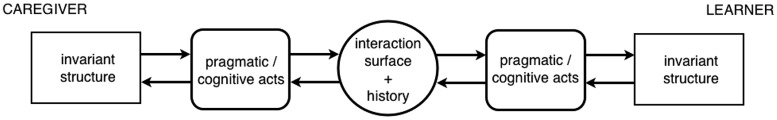
**Pragmatic frames involve operations on the cognitive level as well as on the pragmatic or communicative level (together, they constitute the “meaning”)**. On the cognitive level, we distinguish between (1) perceptual functions and (2) functions pertaining to the representational organization of knowledge. Perceptual functions include low-level processing such as segmentation and classification whereas functions pertaining to the representational organization of knowledge include instantiating a new knowledge item, retrieving or correcting an existing knowledge item. The pragmatic level includes knowledge about the interactional requirement and the pragmatic role of the participants, e.g., that a question is followed by an answer. Interactional experience (history of interaction) is the driving force for deep meaning and deep syntax as constituents of an invariant structure.

We think that one way to grasp this organization of language and action is via the concept of *pragmatic frames.* A pragmatic frame is a negotiated interaction protocol targeted to achieve a joint goal that involves (1) a surface layer, namely, an observable coordinated sequence of pragmatic behaviors in the form of words and actions, (2) a deep structure underlying these behaviors that targets the achievement of one or several joint goals, and (3) a nested cognitive layer that specifies which cognitive operations the frame triggers as it unfolds. We propose that pragmatic frames serve as a communicative foundation or a learning “matrix” (Bruner, 1983, p. 38) that emerges between interactants, and that they are the key to understanding ecological learning processes (see **Figure 1**)

Our proposed alternative to mapping in the form of pragmatic frames is not new and conforms to existing ideas in language acquisition research. However, in this article, we bundle these ideas together, offer new concrete parameterizations that can be used in models of social learning (see Pragmatic Frames—an Introduction and History), and link these ideas more tightly to the current debate on key aspects of word learning (see How Current Approaches Interface with Pragmatic Frames). We hope that our view – though certainly not yet fully developed – can motivate research in language acquisition to study not only individual words but also the emerging action sequences within which these words are crucial for attaining joint goals. We also hope that this theoretical perspective can inspire development in human–robot/(–computer)–interaction, enabling machines to use pragmatic frames and thus to learn and negotiate new interaction structures by themselves.

## Pragmatic Frames—an Introduction and History

What we call pragmatic frames are also known as “interactional formats” (Bruner, 1983, p. 120) or “frames” (Fillmore, 1982, p. 111; Tomasello, 2003, p. 25). Pragmatic frames can be understood as recurrent interactional structures (Bruner, 1983; Ninio and Snow, 1996; Fogel and Garvey, 2007) that emerge over time (see Pragmatic Frames Require a History of an Interaction). In infant development, these structures first occur in a very specific context (Bateson, 1955; Goffman, 1974; Kendon, 1985; Rohlfing et al., 2015). They involve a sequence of goal-oriented actions that is coordinated with the interaction partner (see Pragmatic Frames Involve Goal-Oriented Actions and **Table 1** for more examples). Take, for example, a guessing game in which a child is asked where the lamp is, and she or he points to it. When performing this speech act, a competent speaker knows that the goal has to be framed by a sequence of actions on the surface layer (1) such as looking at the listener and asking a question with a specific prosody and syntax that contains a slot for the requested object. The deep structure (2) involves expecting the listener to manipulate attention (e.g., by a pointing gesture) to make it joint. The cognitive layer (3), in turn, entails the cognitive function for the listener of identifying the requested object.

**Table 1:** Examples of pragmatic frames.

Guessing game (Steels, 2001):
**Speaker**	**Listener**

(1) By pointing, eye gazing or other means, the speaker draws the listener’s visual attention toward an object of interest.	(2) The listener attends to the object

(3) The speaker shares a single predicate that is true for the object of interest but not for the other objects in this context.	(4) The listener shares the predicate and looks up all associations with this predicate in her memory.

	(5) The listener applies the highest scoring association and points to the object of interest.

(6) The speaker gives feedback.	



**Greeting (in which a child learns the name of another person):**

**Caregiver**	**Child**	**Greeted person**

(1) The person recognizes somebody familiar and shares this with the child “Look, there is Anna! Let’s say hello to her!		

(2) Both are approaching the other person or making them visible.		

(3) The person looks at the other person says “hello!” and/or waves.		(4) The person looks up and recognizes a familiar person.

		(5) The listener says “Hello!” to the caregiver and the child or caregiver waves.

(6) Both acknowledge it by smiling.		


**Story telling (Quasthoff, 1997):**

**Story teller**	**Listener**

	[(1) Can ask about an event]

(1) Display of referential relevance	Display of formal relevance (Can ask questions)



(2) Topicalization	

(3) Elaboration	

(4) Closing	

(5) Translation/Evaluation	

	(6) Evaluation



**Action demonstrations (HRI – Akgun et al., 2012):**

**Experimenter/Programmer**	**Teaching user**	**Robot learner**

(1) Start	(2) “New demonstration.”	

	(3) The user moves the arms of the robot and records poses as keyframe with the command “Record frame.”	
	(4) “End of demonstration.”	

	(5) The user can ask the robot to perform the learned movement with the command “Can you perform the skill?”	(6) The robot performs the movement.

(7) End			

Pragmatic frames are retrieved from memory to guide the interpretation of an ongoing situation (see Fillmore, 1982; Bruner, 1983; Tomasello, 2003). Wittgenstein (1953/1997) called such protocols or scripts in which action and language are interwoven “language games [Sprachspiele].” They result in a behavioral disposition within interactants that enables mutual understanding. Language games follow regularities that are constrained by different contexts. Without such regularities, it is not possible for a word to have a meaning.

Steels (2001) modeled language games formally in computational and robotic simulations of the formation of linguistic conventions in groups of agents (Steels and Belpaeme, 2005). In these models, and in particularly in the Talking Head experiment (Steels and Kaplan, 2002), language games allowed robotic agents to successfully negotiate new semantic representations in which words were used as cues to draw the attention of social peers to a shared referent. In such models, language acquisition goes far beyond the mapping mechanism and fits within the pragmatic frame approach we propose here. However, both Wittgenstein as well as Steels and colleagues barely considered the details of the cognitive and developmental dimensions involved in learning these frames.

Schank and Abelson (1977) have addressed cognitive dimensions and emphasized the fact that our interactional knowledge is organized into sequences of actions and linguistic acts. Importantly, this knowledge involves extractions of events “connected directly to the goals and plans to realize those goals made by the participants” (Schank and Abelson, 1977, p. 156). Fillmore (1982) has shown that these extractions comprise semantic roles to evoke the same aspects of a scene in different ways. This knowledge is then used to interpret situations (Fillmore, 1982; see Pragmatic Frames Evoke an Interpretation of a Situation).

A reflection of this frame structure can be found on the linguistic surface in the approach known as Construction Grammar (e.g., Goldberg, 2003). Constructions that relate syntactic structure to meaning are acquired through “repeated exposure to the usage of language in context” (Fischer, 2015, p. 563). Central to this view is, once again, the use of syntactical units that occur in concrete events and then become abstracted. Several computational models, such as Spranger (2011) and Spranger et al.’s (2012) Fluid Construction Grammar, have begun to shed light on how semantic roles evolve and can be negotiated; how particular syntactical units become attached to cognitive operations; or how these links becomes routinized and abstracted through repeated usage (see the usage-based models in, e.g., Langacker, 1987; Behrens, 2009). However, Construction Grammar applies mainly to understanding and acquisition in the context of linguistic interaction. As a result, such frames have hardly ever been included and developed within more general forms of social interaction such as joint actions (but see Dominey and Boucher, 2005).

What interests us is not just the fact that usage-based models allow Construction Grammar to accommodate dynamic aspects of language such as acquisition and change (Croft, 2007). For our approach to what we call the communicative foundation, it is the social-pragmatic aspects of language that are more crucial and the way they preserve a tight link to actions (Harris et al., 1988; Barrett et al., 1991; Tomasello, 2003; Nelson, 2007). By extending the units of syntax to gestures, the concept of pragmatic frames can also be used to refer to the infants’ multimodal ability to enter “into some type of joint attentional focus with a mature language user” (Tomasello and Akhtar, 1995, p. 201). Bruner (1983) describes the value of joint attention extensively, and he ascribes the origins of semantics to the deictic acts of showing and following (e.g., pointing). Tomasello et al. (2005, p. 682) capitalized on this potential of deictic acts revealing that starting from 9 months of age, the child’s ability “to actually share goals” becomes visible when she or he points to, for example, an object: an infant uses pointing gestures, because they are a means to engage the attention of others. Although we think that the ability to engage in joint attention is certainly an important milestone in communication and is achieved not only via gaze and pointing but also via eye–hand coordination (Yu and Smith, 2013) and non-visual modalities (Akhtar and Gernsbacher, 2007), we suggest that joint attention is often a means toward reaching the joint goal (as an element required within a sequence of actions, as noted by Shotter and Newson, 1982) rather than being the purpose of an interaction: Communication with children does not stop at the coordination of attention.

Most recent approaches taking the perspective of a social-pragmatic theory of language acquisition focus on the role of *social cues*. These may be, for example, an eye gaze, a pointing gesture, or a smile. All such cues are supposed to be especially meaningful to young infants (e.g., Szufnarowska et al., 2014) who exhibit a particular responsivity to them (Gergely and Watson, 1999; Senju and Csibra, 2008; Csibra and Gergely, 2009; Csibra, 2010). Whereas some researchers claim that this responsivity belongs to their innate disposition (Csibra, 2010), others provide examples of how this disposition might be educated and emerge over time (Nomikou et al., 2013; Rkaczaszek-Leonardi et al., 2013; Rohlfing and Nomikou, 2014). At this point, we wish to emphasize the difference between the concept of a “cue” and the interactive process of a “co-construction”: whereas a cue would trigger a desired behavior in merely one short moment, a co-construction takes time and requires many turns in a process of *mutual adjustment* (Fogel, 1993; De Jaegher et al., 2010; Rkaczaszek-Leonardi et al., 2014). It is only as a consequence of this mutual adjustment – within which interactants have to exchange behaviors in order to agree on the joint goal (see Dynamic Coupling) – that a behavior eventually becomes a cue. A cue is supposed to direct attention toward a referent (which, in turn, can initiate a mapping). We will argue below that the establishment of a cue is driven by repeatedly occurring joint action sequences.

Whereas the concept of a frame is reduced mostly to particular early games (Bruner, 1983) and social cues in developmental studies, some further specifications can be found in modeling work. Steels (2001) and Steels and Kaplan (2002) (see also **Table 1**) have experimented with various preprogrammed interaction protocols designed specifically to allow robots to learn speech elements (Oudeyer, 2006), lexicons (Steels and Kaplan, 2002), or grammatical structures (Steels and Spranger, 2008). Related work has used similar interactional frames to allow a structured interaction between humans and robots. This has enabled robot learners to identify and learn new elements of language (Roy and Pentland, 2002; Cangelosi and Riga, 2006; Lyon et al., 2012). Work on human–robot interaction has shown that structured interaction protocols based on, for example, mechanisms of imitation or conditioning (Billard et al., 2008; Cuayáhuitl, 2015) allowed users to teach novel sensorimotor skills to robots. However, as we discuss in Vollmer et al. (submitted), the flexibility and power of social learning mechanisms is severely limited. In these existing models (1) the interaction protocols were preprogrammed (i.e., the robot knew how to use and understand them); (2) only few interaction protocols were used at the same time; and (3) they did not include mechanisms to learn and negotiate new interaction protocols. Overcoming these limitations will certainly result in new possibilities of interacting with artificial systems.

Although by allowing rich and varied interaction, protocols may, at first sight, appear to complexify the learning process, the information contained in variable parts of these protocols may actually be the key to cueing the inference process and enabling goal-oriented learning of novel actions in much larger dimensional representation spaces. In the following, we specify the key characteristics of pragmatic frames.

### Pragmatic Frames Require a History of an Interaction

Repetition is central to the power of pragmatic frames. First, the division of roles and tasks is important to co-construct a goal-oriented sequence of social actions. Through the ability to remember a sequence of events (Davachi and DuBrow, 2015), its repeated occurrence enables participants to develop expectations regarding how actions relate to each other (Marcos, 1991; Nomikou et al., 2016, accepted). Repeating a particular situation leads to a familiarization effect. A familiarization results in (1) easily interpretable settings, and (2) lower cognitive load (Bruner, 1983). In the following, we explain these two strongly related effects in more detail.

In their first and second years of life, children learn primarily on the basis of familiar elements in the context, and they need environmental or social support to produce the appropriate behavior. Some studies have shown that elements of an ongoing situation give rise to cognitive operations in infants. Recently, for example, Parise and Csibra (2013) showed that children exhibited an N-400 semantic priming effect only when they heard their mothers voicing words referring to a visual stimulus presentation. With respect to children’s non-verbal behavior, Beisert et al. (2012) found that facing an unusual situation hindered imitation ability in 14-month-olds.

Marcos (1991) has cast light on the process of social support in repeating contexts and its value for language acquisition. In this study, mothers described a poster repeatedly to their 12- to 13-month-old children. Results showed that in comparison to controls, infants in the repeating context condition showed an increase in (1) the time devoted to referential behavior, (2) the number of infant initiations of dialog, and (3) the use of pointing gestures.

Farrar et al. (1993) examined the value of the familiarity of situational aspects for processing capabilities by observing mothers and their 2-year-old children playing with toys in either a familiar setting (same toys in all sessions) or an unfamiliar setting (new toys in each session). Comparing children’s word productions across settings revealed that children used more different lexical types, used more verbs, and had a higher mean length of utterance in the familiar toy setting. Farrar et al. (1993, p. 603) suggested that the familiarity with the toys provided both “a conceptual framework for interpreting the event” and an increase in “processing space.” In this sense, a familiar situation cues the retrieval of the appropriate meaning, because highly frequent items form stronger associative networks than less familiar sets (cf. Bjorklund, 1987). In another study, Rohlfing (2006) investigated the acquisition of spatial prepositions in 2-year-olds by presenting them with sets of toys that were either familiar or unfamiliar. Results showed the best learning with familiar sets. Furthermore, familiarity not only seems to support the interpretation of an ongoing situation, but may also be a prerequisite for the extension process by which children generalize their knowledge to novel situations. Without the ability to apply what they have learned in a familiar situation, children would be unable to generalize a spatial preposition to novel objects. This seems to indicate a hierarchical structure of learning proceeding from a familiar context to the ability to generalize (Rohlfing, 2006).

Taken together, this evidence suggests that the familiarity of a situation influences the perception of the overall interaction setting and the required degree of effort. When interpreting a situation, children need to hook up with familiar elements.

Currently, we do not know on which basis children are able to recognize a repeated situation, thereby enabling them to anticipate actions (Schacter et al., 2007; Ramscar et al., 2010). The ability to recognize sequences of events certainly plays a key role. In fact, in recent neurophysiological approaches, Davachi and DuBrow (2015) showed how sequences elicited hippocampal patterns that stabilized through repetition. They suggested that this “may be a distinction between single-trial or episodic sequence encoding and the representation of a well-learned, repeated, predictable sequence because each re-exposure to a sequence may modify the learned representation” (p. 2). This distinction also needs to be studied in developmental research. Although the comparison of learning situations is already recognized as a powerful mechanism (Yu and Smith, 2007; Trueswell et al., 2013), its use has been limited to investigations across trials or to fixed delays (e.g., Vlach, 2014). We need to fill this research gap and explain the process of dynamic integration between learned regularities and the immediate contextual cues guiding attention at the specific moment (Smith et al., 2010) that yields “attentional hierarchies” (Bahrick and Newell, 2008, p. 993).

### Pragmatic Frames Involve Goal-Oriented Actions

Bruner (1983, pp. 24–31) acknowledges that children are equipped with some predispositions that help them to make sense of recurrent situations. He calls this the “initial cognitive endowment.” One proposed prerequisite is that children transfer experiences into means–goal structures (p. 18). This endowment supplies some semantic targets (p. 34). Similarly, Nelson (1974) proposes that actions and their results are at the core of children’s concepts (see also Mandler, 2012). In fact, in their early infancy, humans are “obsessed” with goals. Csibra and Gergely (2007) propose two main epistemic functions that this obsession serves: online prediction (see below) and social learning.

*Online prediction* encompasses the fact that infants look at (Baldwin et al., 2001), imitate (Meltzoff, 1995), and anticipate (Woodward, 1999; Falck-Ytter et al., 2006) others’ action goals rather than regarding other aspects of observed behaviors. Several researchers assume that the basis for this is the way the infant’s own motor system develops while gathering own motor experiences (Woodward, 2009; Kanakogi and Itakura, 2011). Thus, when observing someone performing an action, children will simulate an action plan covertly (Rizzolatti et al., 2001) if this plan is already in their action repertoire (Woodward, 1999).

Csibra and Gergely (2007), in turn, offer an alternative approach to action understanding in which situational constraints are relevant. “Teleological reasoning […] requires the recruitment of the relevant background knowledge that the observer accumulated about the physical constraints of the situation and of the actor” (Csibra and Gergely, 2007, p. 70). This argument is supported by the fact that infants attribute goals not only to conspecifics but also to abstract and artificial agents.

Yet another source that might serve as a database for infants to recognize a structure in the relation between an involved agent and her or his goal is the interaction (Reddy and Uithol, 2015; Nomikou et al., 2016, accepted). Even though Gerson and Woodward (2014) have argued recently for a “unique effect of active over observational experience” on the recognition of goal structures in actions, this effect might appear only because of the dichotomy between active and observational experience. But what about an experience in which an infant is only a part of an action? Reddy et al. (2013; see also Lock, 1978) have shown impressively that 2-month-old infants already perform anticipatory adjustments of their own body when they see a caregiver approaching to pick them up. Clearly, infants learn very early on in their development that one of the most important means to achieve their own goals is the actions of their caregiver. Simultaneously, they experience their own actions in the light of goals as interpreted by their caregivers: Rkaczaszek-Leonardi et al. (2013) have shown that seemingly random movements of 3-month-olds will be interpreted as some form of collaboration and weaved into a joint activity. It should be noted that the notion of goals, and especially how goals are represented, is currently underspecified in the scientific debate (Wrede et al., 2012). More specifically, little is known about bottom–up ways for learners to infer or detect the (potential) goal of an action. Wrede et al. (2012) argue, however, that it is reasonable to assume bottom-up biases that exist and predispose the learner to identify goal-relevant features. The relevant definition of a goal should also encompass the social dimension. Little is known, for example, about the role of emotional attunement in goal recognition, although it has been recognized as being crucial in infant development (Legerstee, 2005; see also Stephens and Matthews, 2014, for a brief review). Combining emotions with action goals, Rossmanith et al. (2014, p. 8) have proposed that “action arcs” shape activities with children. These are built up within the flow of the interaction and thus consist of a beginning, a building up, a climax, and a resolution. The term “climax” is similar to our conception of the joint goal but pinpoints the emotional function that sequentially organized actions have to fulfill.

#### Social Learning

Children experience goals by being active, by being a part of a collaborative activity, and by being interpreted as active. In fact, the goal of a joint interaction is central to the idea of the pragmatic frame studied in this article. We conform with Tomasello et al. (2005, p. 676) in believing that “human beings […] are biologically adapted for participating in collaborative activities involving shared goals and socially coordinated action plans.” With growing interactional experience, children become able to elicit goals on their own. However, we hypothesize that they start with ideas of goals that differ significantly from the goals pursued by older children or adults. In this sense, inducing a significant change in the environment such as turning the light on and off might well be an attractive goal for a young child (Wrede et al., 2012). Such goals are commonly used in the first steps of language acquisition: Stern and Stern (1975) reported a first understanding in their child following an instruction to change the environment in a significant acoustic way by, for example, ringing a bell or clapping hands. Nelson (1974, p. 279) also remarks that early vocabularies include objects that “move or change in some way or that child can act upon” so that the goal of the activities is perceivable.

This vision of how the acquisition of new words can occur as a side effect of interaction games leading toward joint actions leads us to disagree with Tomasello’s (2001) suggestion that language acquisition is possible only when children develop the skill of joint attention. We think that joint attention is helpful—and, as a frame, it massively boosts word learning. However, we think that children gain a rich interactional experience in expecting and coordinating actions that is crucial for language learning from other frames consisting of goal-directed actions in a sequence in which roles have to be fulfilled and children’s attention is educated in the sense of fulfilling this role (not necessarily visually). The goals of these actions are crucial for their organization, and we locate these on the deep structure level (see below). Our view is supported by Mastin and Vogt’s (2015) comparison between urban and rural communities in Mozambique. They found that language is unlikely to occur during joint attention with objects in a non-industrial rural environment in which object stimulation is not common. Instead, the vocabulary scores of children raised in this environment correlated positively with dyadic activities engaged in through touch and ritualized play. Here, further cross-cultural studies are necessary to reveal alternative interaction protocols. Along these lines, Nelson (2007, p. 118) suggests that even in societies in which parents do not engage in naming things, “immersion in a language-using community” contributes to patterns of interaction. These patterns are pragmatic frames that enable children’s learning.

### Pragmatic Frames Consist of a Meaning and a Syntax

Bruner (1983, p. 46) points out that pragmatic frames can be characterized by a surface and a deep structure: he uses *deep structure* to characterize the invariant basic form of a pragmatic frame and *surface structure* to characterize its appearance (i.e., the variable forms in which the deep structure is realized). For Bruner’s (1983) famous example of the peek-a-boo game, the deep structure is about hiding and reappearing, whereas the means for it can vary on the surface structure.

We prefer to account for pragmatic frames by using the terms **meaning** and **syntax**. This allows us to further differentiate the structure introduced by Bruner (1983) (see **Table 2**). Another crucial difference is our inclusion of cognitive operations among the ingredients of meaningful behavior that are needed for an appropriate coordination and disposition of the participants.

**Table 2:** Our concrete parameterizations of pragmatic frames.

	Syntax	Meaning
Surface	– External	– Hidden
	– Directly observable	– Cognitive operations recruited from memory
	– Sequence of behaviors	
	– Execution of deep syntax	– Emerging cues of behavior
Deep	– Invariant properties inferred from, e.g., statistical learning	– Hidden
	– The basis for a sequence	– Constructed around joint goal(s)
	– Specifies slot and type of learning content	– Cognitive operations in a sequence for a (joint) goal
		– Long-term effects on memory
		– Specification of learning content possible because it is embedded in familiar goal-directed sequence of actions

#### Meaning: Connection to the Cognitive Processes

We call the set of effects that a frame has on memory processes (i.e., the cognitive functions involved in the frame) the **meaning** of a pragmatic frame. Whereas, of course, memory effects are present for both interaction partners (see **Figure 1**), we focus on the learner side. In line with Bruner’s (1983) surface and deep structure, we differentiate the *surface* from the *deep meaning* of a pragmatic frame. The *surface meaning* denotes the set of cognitive operations associated with the pragmatic frame. We presume that these emerge with a child’s growing interactional experience. For example, Nomikou et al. (2013) showed that mothers use sensitivities toward motion and intermodal synchrony to educate their infant’s gaze behavior, and that this develops into a convention. As an effect, gazing at the interaction partner communicates a referential expectation (Senju and Csibra, 2008; Szufnarowska et al., 2014). The surface meaning consists of such conventionalized signals; that is, individual elements of behavioral patterns (pragmatic acts) that are known to the learner from previous interactions, already acquired pragmatic frames, or constituents of such. They can be basic and automated in terms of being reactive behaviors, but bear some dispositions that are co-constructed with the partner: for example, when a tutor points to an object, the learner not only follows the gesture but also expects a referent (Gliga and Csibra, 2009). These operations do not necessarily appear constantly, but may vary to a similar degree as the constituents of the surface structure. Take, for example, the way blind people perceive a new object via touch or hearing (Bigelow, 2003). Although this way will differ in the type of cognitive operations needed to process the perception, it results in the same deep structure. The patterns of the surface meaning thus create anticipation of the learning content on the cognitive level.

We use the *deep meaning* of the pragmatic frame to refer to the goal-directed cognitive operations involved in the processing of, for example, its *learning content*. The deep structure differs from the surface structure in that learners will experience more interactions and more variability in the performance of the behavioral sequence. As a consequence, they will be able to extract the invariant regularities and generalize the interaction structure. This extraction might be a kind of embodied abstraction (Binder and Desai, 2011) from concrete modality-specific memory to amodal memory. Although the understanding of the generalization process is still nebulous, we believe that it produces a kind of schema/construction (Nelson, 1974; Behrens, 2009; Binder and Desai, 2011). Constructions make a slot available to users that can take different forms: a non-verbal behavior within joint engagement (Nelson, 2007) or creative verbal behavior (Lieven et al., 2003). That is, children can expect a particular form of interaction (see also the deep syntax for more details), because the deep syntax allows them to tune into the content. For example, in a labeling frame, a learner will expect that the utterance “this is a...” performed with a pointing gesture toward an object is about associating a word with this object. In this frame, the learner’s role is to follow the gesture, extract a single object from the complex scene, and memorize its label in order to recall it when the referent is present. Thus, the learning slot affects memory but is linked to the pragmatic role that the learner takes in this frame. In other words, gaining a grasp of the deep meaning of pragmatic frames is equivalent to knowing what the interaction is about (Lock, 1978).

Thus, cognitive skills are linked to pragmatic skills as an important and innovative aspect of the pragmatic frames concept proposed here (see **Figure 1**). With pragmatic skills, we refer to children’s ability to contextualize their (verbal and non-verbal) behavior for a specific purpose. We predict that training with pragmatic frames should lead to an improvement in related cognitive skills that can be assessed by testing slow mapping abilities (in an interactive setting with an experimenter) and language processing (in an eye-tracking setting). Thom and Sandhofer (2009) have already provided some support for this prediction by demonstrating that 20-month-old children learned color words more robustly when exposed to more instances during training. We assume that the training in this study advanced the children’s ability to prioritize the colors of the objects in their memories.

For the meaning of pragmatic frames in general, we hypothesize that not all aspects within a frame will be learned at once, but that learning will proceed from the surface level (because recurring, directly observable patterns are easier to identify) to the deep level and from the syntax level to the underlying cognitive level. This indicates the need for different attentional processes (i.e., from surface to cognitive level) (Krauzlis et al., 2014). For example, turn taking is learned very early in a child’s development (Kaye and Wells, 1980) and enables a child to develop such cognitive operations as eliciting, expecting, and waiting for somebody’s response. These operations can be used on a surface level as a first encounter with the meaning. Our hypothesis is that observations of productive behavior (elements of the surface level) might be acquired before an understanding of behavior (operations needed on the cognitive level) in situations in which communication is scripted and thus transparent. In fact, Salas Poblete (2011) showed that in highly structured triadic situations in which they could imitate a model, 2-year-old learners displayed a productive communicative behavior (an appropriate gesture for a referent) without grasping the underlying robust meaning of a pragmatic frame that would allow them to transfer it to a new exemplar of the referent.

#### Syntax of Pragmatic Frames

We will call the sequence of verbal and non-verbal actions that characterize the appearance of a pragmatic frame its **syntax**. Like Bruner (1983), we define the *surface syntax* as the observable sequence of behaviors constituting the pragmatic frame. The surface syntax comprises, amongst others, the adequate sensory means, the possible orders of behavioral units, and information about actors. Surface syntax also specifies the sequence of cognitive actions. We assume that such cognitive operation sequences need to be learned (and automatized). One example of how a sequence can change the subsequent operations is given in Moll et al. (2006). In this study, children at the age of 14, 18, and 24 months witnessed an adult expressing excitement while looking to the side on which an object was located. In a condition in which the object was new for the adult, children responded by attending to the whole object. In a condition in which the child and the adult had previously played with the object, children responded by attending to a specific part of the object or by expecting another object of interest to be present in the room. Clearly, this behavior is not a part of the operations and roles related to the common frame in which a learner encounters a new object.

For word learning, deep syntax can specify the slot for the (learning) content (i.e., where it is in the sequence) and the type of content (e.g., whether a noun or a color is learned). The function of deep syntax is twofold: first, it reflects the generalizability of the interaction structure (because the invariant parameters will be gathered across various experiences); second, it contains information about the (learning) content and thus what the sequence is about—this constitutes a link to the deep meaning (see above).

Take the action of pointing to an object and labeling it. Performing this act, a competent speaker knows that its goal has to be framed by (1) looking at the other person, (2) pointing in the direction of an object, and (3) uttering its label. The role of the learner is then to recall this label in the presence of the referent. When learning new words, this sequence will be repeated with varying content in only some slots. Thus, the learning dimensions within frames are limited to operations such as to identifying the slot within a known frame and to processing the new information in the slot leading to a knowledge update.

Crucially, the slot with the learning content (e.g., a new word) works successfully only when the learner is able to apply the content (e.g., a new word) appropriately (e.g., by choosing the correct object from an array). Once cognitive operations are set up in line with a collaborative behavior for a specific learning task, the changing elements likely become easy to pick up. Note, however, that the element will be picked up only because it is crucial for the joint goal (e.g., the tutor expects the learner to choose the correct object and to give it to the tutor). The (learning) content, which is put into a particular slot, links the deep syntax to the deep meaning.

For learning, the relevant content is given by the deep level of both syntax and meaning. Whereas a pragmatic frame that appears to be situated solely on the surface level does not seem to contain a slot for learning in the explicit learning sense (compared to, e.g., the frame of greeting someone), the learner can prescind from this frame to create a slot for learning—for example, whom to greet and whom not to greet. Clearly, we need to specify which circumstances can push the learner forward: Is it the tutor, the cross-situational comparisons, or the negative examples? We presume that not only the deep meaning but also the deep syntax are involved in the schematization process (Behrens, 2009; Fischer, 2015) that results in abstract representations. This idea is compatible with Nelson’s (1974) suggestion that the functional core of a concept for individual words becomes synthesized from concrete acts and their various relationships. It is also compatible with Barrett et al. (1991) multiroute model of early lexical development. They suggest that context-bound words (initially mapped onto an event representation) and social-pragmatic words might be learnt differently from referential words such as nominals or non-nominals (initially mapped onto a prototype). We think that a frame of use for these words may well influence the kind of representations that arise. A concrete examination of word learning frames might help to verify this theoretical assumption. Roy et al. (2015) have suggested that in order to acquire verbs (in contrast to nouns), children might need the support of the activities taking place in a particular space.

Different frames might result in different forms of representations. Input can certainly help children to organize their knowledge (Gelman, 2009) and to exercise cross-situational comparisons (Gelman et al., 2005). In addition, the syntax of a frame can vary to some extent from person to person. Thus, children organize their conceptual knowledge on the basis of private experience that is “different from the adult’s and from the conventional meaning” (Nelson, 2007, p. 124). The form of the parts that constitute the syntax varies according to the utterances and tokens used (e.g., when uttering the label in the pointing–labeling frame described above, possible tokens include X [ = label], it’s an X, that’s an X, there’s an X [cf. Bruner, 1983, p. 79], intonation, prosody, and pause lengths). Young infants, however, are presented with a stable caregiver’s behavior on which they can rely. Certainly, the syntax of a pragmatic frame is highly conventionalized and “cannot be specified independently of the perceptions of the participants” (Bruner, 1983, p. 133). We assume that children need to experience a lot of variations on the surface level to grasp the deep meaning. The role of variation is compatible with what Clark (1993) proposes as the principles of contrast and conventionality that guide children’s word learning. We view pragmatic frames as interaction protocols that eventually become conventionalized. And when words are applied in different frames, these contrasts might advance their schematization (see above).

### Hierarchy of Pragmatic Frames

Currently, one of the major challenges in this alternative approach is to determine how far formats may be modular; that is, how far they may be composed to form bigger units or recomposed to form other sequences. Can a pointing gesture to an object already be a pragmatic frame? Or is it just a part of it? Humans have the capacity to assemble a new structure from previously experienced elements (Davachi and DuBrow, 2015). Hence, it is likely that pragmatic frames can be created *ad hoc* from known elements. Heller and Rohlfing (2015) found that children as young as 13 months create new gestural practices when narrating a picture. Yet, this novelty occurred in the familiar context of joint book readings. For verbal behavior, Lieven et al. (2003) showed how a 2-year-old child was able to perform some operations (such as substitute, add-on) on previously heard utterances.

Bruner (1983) recognizes that:

Formats are also modular in the sense of being accessible as subroutines for incorporation in larger scale, long-term routines. A greeting format, for example, can be incorporated in a larger scale routine involving other forms of joint action. In this sense, any given format may have a hierarchical structure, parts being interpretable in terms of their placement in a larger structure. The creation of higher-order formats by incorporation of subroutine formats is one of the principal sources of presupposition. What is incorporated becomes implicit or presupposed (p. 133).

Along these lines, we agree with Bruner (1983) that:

Formats “grow” and can become as varied and complex as necessary. Their growth is effected in several ways. They may in time incorporate new means or strategies for the attainment of goals, including symbolic or linguistic ones. They may move toward coordination of the goals of the two partners not only in the sense of “agreement,” but also with respect to a division of a labor and a division of initiative. And they may become conventionalized or canonical in fashion that permits others within a symbolic community (e.g., a “speech community”) to enter the format in a provisional way to learn its special rules (p. 132–133).

Nonetheless, the challenge is still to create a model that can grow by (1) accepting new means for the effects, (2) modulating goals, and (3) accepting new frames when they become conventionalized, that is, a part of an interactional routine.

### Pragmatic Frames Evoke an Interpretation of a Situation

To date, investigations of pragmatic frames have concentrated on whether and at which age children master a particular routine. For example, Franco and Butterworth (1996) have shown that 16-month-olds have already learned to visually check whether their interlocutor is attentive before they point to something. van der Goot et al. (2014) conducted a series of experiments showing which conditions have to be fulfilled for infants to point and what children expect their partners to do in a particular context (Thorgrimsson et al., 2014, 2015). This research attests to conventionality and children’s growing expectations that communicative situations will conform to a particular structure. Children expect a structure not only on a syntax level but also on the level of meaning. Matthews et al. (2010) showed that children usually expect only one label to refer to an object. They broke these “referential pacts” (Matthews et al., 2010, p. 749) by having different experimenters assign different labels to the same objects. When responding to the new labeling, 3-year-olds were slower, suggesting a greater cognitive load. Metzing and Brennan (2003) found that children demonstrated social-cognitive abilities unlike those of adults because they expected referential pacts to persist across experimenters. Some children protested explicitly: “While they understood that the alternative terms were intended to refer to the same object, they were very keen to pass normative judgment on term use and did not appear to fully appreciate that different people might take different perspectives on an object” (Matthews et al., 2010, p. 756).

In our opinion, previous experimental research has focused on very specific pragmatic frames (mostly labeling) and described their functions for a specific kind of learning. What is still lacking, however, is a broader perspective that accounts for other learning situations (Nakao and Andrews, 2014) and thus other possible pragmatic frames. One interesting recent study was conducted by Moore et al. (2013). They presented 3-year-olds with a hiding game and showed that the children could comprehend a novel communicative act even without the means of communication on which they typically rely. However, the children seemed “to exploit a number of everyday bodily cues in interpreting communicative intent” (Moore et al., 2013, p. 75). Thus, apparently, children are on the lookout for familiar frames that may help them to interpret an ongoing situation.

One of our studies (Salas Poblete, 2011; Rohlfing et al., 2013) provides a first methodological approach for manipulating pragmatic frames actively in the context of word learning in order to explore their influence on learning success. Specifically, we tested children’s word learning in known labeling frames and in frames consisting of new elements. For the latter, the object was highlighted by a light being switched on under it instead of using the familiar pointing gesture. We found that children still learned new words in unfamiliar frames (Salas Poblete, 2011), suggesting that new elements might slow down but not eliminate learning. This implies that young learners might be tolerant toward changes in the elements of a frame. It remains unclear whether some elements (e.g., ostension) can be interpreted more strongly as a particular frame than others (e.g., pointing).

Many open questions arise from these considerations: If a child needs to experience a situation repeatedly to accumulate information about it, we need to know what aspects of a situation are crucial for learning and whether children differ in the way they *construct* a situation. Crucially, we should look at the entire situation as constructed by individuals and not at isolated aspects of it. The first step could be to identify whether and how significant changes to the learning situation impose a greater cognitive load on learners. Because the interactive roles in a particular situation seem to be crucial for establishing a pragmatic frame (Bruner, 1983), it is relevant to investigate whether children’s perception of a situation changes when they are able to reverse the roles (Carpenter et al., 2005). In a next step, future research needs to explain how a *change* of a frame can be detected. A solution is necessary to account for the question regarding which factors indicate that a new frame is initiated and, thus, to allow intelligent systems to act flexibly within an interaction (Vollmer et al., submitted).

### Asymmetric Pragmatic Frames: Scaffolding

For language acquisition, it is important to consider the frames that caregivers establish intuitively when eliciting and training specific behavior in children. More specifically, a caregiver seems to reduce “degrees of freedom in the task to manageable limits” and to mark critical features (Wood et al., 1976, p. 99; see also Pitsch et al., 2014). Bruner (1983) recognizes that:

One special property of formats involving an infant and an adult […] is that they are asymmetrical with respect to the knowledge of the partners—one ‘knows what’s up,’ the other does not know or knows less. Insofar as the adult is willing to ‘hand over’ his knowledge, he can serve in the format as model, scaffold, and monitor until the child achieves requisite mastery. (p. 133)

Following the “interactive turn” in social cognition research (De Jaegher et al., 2010; Cowley, 2011), recent studies have revealed that an unfolding interaction provides the tutor with an opportunity to adapt to the learner’s needs over time (e.g., Fukuyama et al., 2014; Pitsch et al., 2014). Continuing this line of research, we propose that modifications by the more competent partner offer the learner the possibility to join the interaction, even though the learner might not understand every detail of it (Wrede et al., 2013). Yet, we know little about how children gain a grasp of the deep structure when acting on the surface. Future research needs to examine how far some pragmatic frames are more appropriate and efficient for training than others: In what way might their structure be more transparent than the structure of other frames? In a study with 14- to 18-month-olds, Rohlfing et al. (2015) found that specific types of pragmatic frame such as labeling and questioning routines occurred in the context of joint book reading. Importantly, both frames fostered the child’s participation in an interaction involving pointing and later answering questions and were related to children’s later vocabulary. Although also observable in other contexts such as free play, the context of joint book reading seems to richly elicit specific kinds of frames (Gelman et al., 2005; Rohlfing et al., 2015). We need to understand more about deep syntax in the sense of the cognitive operations that give rise to subsequent sequences of actions.

### Individual Differences

Because pragmatic frames comprise a link between communicative and cognitive skills, individual differences might emerge in these skills (Akhtar and Gernsbacher, 2007) and in what individual exposure a child needs to learn a frame: some children may establish some kind of structure after only a few exposures, whereas others will need more repetitions to take advantage of the frame (Rohlfing et al., submitted). The literature reveals other examples of the bandwidth of important aspects when learning frames. One essential aspect has been reported by Bedford et al. (2013), who gave word-learning tasks to 2-year-old toddlers at high and low risk for autism spectrum disorders. One group of children received social feedback confirming their choice; the other group received no feedback. Results showed that children with a low risk for ASD benefited from feedback on their initial choice, and that their further performance was above chance level. In contrast, those with a high risk for ASD did not show this effect. Because their pragmatic competencies are commonly viewed as being impaired, it is possible that they have difficulties in (1) recognizing a sequence of actions as a whole/frame or (2) prioritizing information within the single elements of a frame (giving higher priority to the confirmation rather than to own choice). Future research should investigate how individuals may differ in picking up a structure and learning from it.

## How Current Approaches Interface with Pragmatic Frames

Even though many current approaches interface with the idea of pragmatic frames, most of them consider the brief moment of learning but not the history of interaction. As a consequence, these approaches explain only some surface aspects of learning and do not account for the communicative foundation underlying language acquisition. In the following, we examine how some existing perspectives on language learning interface with our approach. This allows us to provide further details on pragmatic frames and contrast them with existing concepts.

### Social Cues

Whereas many recent studies advertise the role of social cues in the process of mapping a word onto its referent (Horst and Samuelson, 2008; Axelsson et al., 2012), strong criticism of the mapping metaphor can be found at the core of social-pragmatic approaches (Nelson, 2007). Tomasello (2001) explicitly suggests dropping the mapping metaphor in favor of a referential area. He attributes the learning process not to two independent entities, namely, the word and its referent, but to a child who is analyzing the whole situation in terms of a joint action goal. This situation analysis focuses on a *person* using a symbol to manipulate the other’s attention.

Despite cogent reasons for regarding the continuation of actions rather than just a moment as contributing to language acquisition, we think that this latter focus has its own justification, because adults can shape their vocal and non-verbal actions into “categorical units of cultural communication” (Fogel, 1993, p. 29). For an adult, a particular element, such as an eye gaze, a word, or a vocalization with a particular prosody bears a potential meaning. Thus, it is likely that when learning language, children progress from utilizing larger behavioral units to short sequences/cues in order to ascribe meaning in communication. This idea is supported by studies showing that children *can* already recognize single elements (previously seen within a sequence of actions) and interpret them in accordance with their experience. One remarkable example is Senju and Csibra’s (2008) study revealing that 6-month-old infants can already interpret eye gaze as a cue signaling a reference to objects. Children probably become educated to such cues (Nomikou et al., 2013) and can take advantage of them in later learning processes. This advantage has been demonstrated convincingly in Horst and Samuelson’s (2008) study showing how social cues in the form of an ostensive labeling can influence children’s learning performance. Children at the age of 24 months were presented with novel objects in either an ostensive (addressed and being gazed directly when labeling an object) or a follow-in naming condition (the object was labeled while the child was manipulating it). After 5 min delay, they could remember new labels presented in the ostensive but not in the follow-in naming condition. We argue that this ostensive condition takes advantage of a labeling frame that seems to trigger some specific cognitive operations in children (long-term remembering). However, the authors themselves interpreted their findings in terms of situational cues guiding the child’s attention and memory.

The literature on cognitive and language development reveals a consensus that in natural settings, children aged about 18 months can robustly figure out an intended referent on the basis of joint attention (and some cues) even when some noise is interfering with the situation (Baldwin, 1993; Carpenter et al., 1998). What is controversial, however, is the role of social information before the age of 18 months, and at which age children become sensitive to the social information that is considered to be a means of attaining joint attention in the form of eye contact, gesture, and language.

Pruden et al. (2006) tested which information – social or perceptual – 10-month-old children would apply to associate a word with an object. Whereas perceptual information was operationalized as the salient appearance of the object, the direction of eye gaze stood for the social information. The authors found that at the age of 10 months, infants regard the salience of objects rather than the eye gaze, and they concluded that perceptual but not social cues are weighted more heavily at this age. Applying Pruden et al.’s (2006) findings in an emergentist coalition model (Hirsh-Pasek and Golinkoff, 1996; Hollich et al., 2000; Golinkoff and Hirsh-Pasek, 2006, 2008) encompassing different sources of knowledge in the process of reference resolution, indicates that when it comes to word learning, social perception as a skill is acquired later in development (see also Booth et al., 2008). For younger children, general learning factors such as the sensitivity to perceptual salience seem to be more important. We think that the critique of social cues is justified if individual cues are expected to control attention and memory. However, cues do not occur in isolation in natural interactions, but embedded in a sequence of actions and together with other cues. This provides a rich environment, and attention and memory processes take advantage of the unfolding situation—and do not just start at the moment when the word is uttered.

Flom et al. (2004) have shown cogently that when cues were presented to in 9-month-olds in combinations (which is the case in natural settings), the frequency of gaze following toward peripheral targets increased. Hence, it is likely that additional cue alongside or preceding the eye gaze presented by Pruden et al. (2006) would have guided the children’s attention to the referent more reliably than a single cue. Clearly, different timescales have to be taken into consideration (Rkaczaszek-Leonardi, 2015): the immediately preceding context *and* the interaction experience that the child brings into the situation. Concerning the immediate context, Liszkowski (2014) recently highlighted the importance of preceding action contexts as a source of symbolic development. More specifically, he reported findings revealing that in 12- to 14-month-old children, the outcome of the reference process differs depending on what the agent has done or seen before (see also Moll et al., 2006). Thus, joint actions are crucial and establish a context/history of interaction against which young children already interpret or use the communicative means. Whereas Liszkowski (2014) discerns the immediately preceding action contexts as an ingredient of meaningful behavior in young children starting to communicate, he barely considers the conventionalization process (Clark, 1993; see Pragmatic Frames—an Introduction and History). Waxman and Gelman (2009, p. 261) made the critical point that associationist approaches disregard “the fact that each word participates in an exquisitely detailed linguistic, social, and symbolic system.” It is not just the association that is formed in the learning process. Instead, children learn to apply particular cognitive operations (some cognitive and communicative jobs) in coordination with their partner. In other words, social cues are not only a part of the immediate context but also a part of the physical events that the child has already experienced with another person in the past. They are, therefore, parts of events with a specific interaction history (Rkaczaszek-Leonardi, 2015, p. 7).

Indeed, studies with young infants show convincingly that language as a signal possesses a unique power from early on: Words, and not just tones, induce categorization processes in infants as young as 3 months of age (Ferry et al., 2010). It is reasonable to think that the link between speech and the fundamental cognitive process of categorization might well be part of an innate endowment. However, recent research suggests that the link might be based rather on the communicative patterns – what we referred to above as communicative foundation – that the children have already experienced: In a recent study by Ferguson and Waxman (2016), 6-month-old infants were presented with videos containing interactions between persons who used ‘beeps’ to communicate with each other in a contingent way. After this exposure, infants were then tested on whether these beeps facilitated the categorization of objects. The authors found that 6-month-olds can apply an otherwise non-communicative signal to categorize objects if they experience this signal in a cooperative, turn-taking setting. We think that these findings taken together support our argument that a communicative foundation is necessary to then give rise to communicative means that are connected to cognitive operations.

### Dynamic Coupling

With respect to the different timescales that give rise to meaningful behavior in communication, current pragmatic theories (Schumacher, 2014) suggest that a phase architecture drives a communicating system. Whereas in the first phase, as suggested above, the system is supplied with cues that help to generate expectations for upcoming words and actions, in a second phase, a representation can be updated by dynamic coupling known also as alignment. Beyond developmental research (see Stephens and Matthews, 2014, for a brief review), the process of alignment between interactants is known in studies with adults (Pickering and Garrod, 2004, 2013; De Jaegher et al., 2010). According to the interactive alignment account, the automatic process of alignment is observable in the verbal (Garrod and Anderson, 1987), gestural, and non-verbal (Kimbara, 2006; Bergmann and Kopp, 2012) behavior of interactants and results in aligned linguistic representations. For language acquisition, this perspective implies that interaction should not be investigated as a “mere context” in which learning takes place; instead, interaction should be seen as a part of the cognitive processes (Nomikou, 2014, pp. 43–44) allowing individuals to coordinate their cognitive operations to achieve a joint goal. Investigating learning within interactions means modifying the idea of how individual cognitive mechanisms work (Nomikou, 2014). Hsu and Fogel (2003) propose considering mother–infant dyads as a whole rather than separating them into individual participants. In fact, this unique coupling of the caregiver with the learner gives rise to observable phenomena in child-directed behavior: Visible as caregivers’ child-directed behavior, these modifications have been observed in speech (Fernald and Mazzie, 1991; Dominey and Dodane, 2004), gesture (Iverson et al., 1999; Grimminger et al., 2010), and motion (Brand et al., 2002; Rohlfing et al., 2006; Wrede et al., 2013). Their function has been appraised across disciplines as facilitating children’s recognition of the structure in language and action. The first approaches toward a social feedback loop are now being formulated (Fukuyama et al., 2014; Pitsch et al., 2014; Warlaumont et al., 2014). However, further theoretical developments need to acknowledge that caregivers’ responsiveness is not restricted to particular cultural patterns such as joint attention but rests upon joint action— as already anticipated by Shotter and Newson (1982).

### The Importance of Statistical Learning and an Accumulating Linguistic Knowledge Base

In Section “Pragmatic Frames Require a History of an Interaction,” we emphasized that pragmatic frames allow us to capture different time scales. The value of information accumulating within and across situations has been recognized by some authors who propose that children do not need a conceptual representation to map a word onto its referent in specific situations. *First*, grounded in the sense of novelty, an association can be established by the child’s perception of a novel object that will stand out because it is unfamiliar (Mather and Plunkett, 2012). *Second*, children benefit from their memories across situations, and they notice which elements remain constant across multiple uses of a word and thereby ascribe a meaning to it (Akhtar and Montague, 1999; see Smith and Yu, 2008; Yu and Smith, 2012, for computational models on cross-situational learning). The ability to compare across situations is in line with Bruner (1983) who emphasizes that children’s sensitivity to invariant aspects of a situation might be a part of their cognitive endowment. Even though children’s ability to compare across situations is viewed as crucial, there are only very few models that try to explain how children integrate these experiences with online information. Although McMurray et al. (2012) provide a model that uses two timescales, this is restricted to the one frame of learning the meanings of novel words.

Certainly, more flexible models are needed that can account for the integration of timescales without being limited to one task. In addition, models need to account for the fact that children seem to differ with respect to their ability to recognize invariant aspects of a situation. One excellent example of this individual difference is the shape bias. Jones and Smith (1993, p. 132) argued that shape bias emerges as “the child learns to represent the regularities that exist between how words are used, the co-occurrence of properties in objects, and the act of attending to particular properties.” Interestingly, children who are at risk for delay in language acquisition, so-called late talkers, seem to gain a different operation from their word-learning experience than their age-mates and lack “a potentially helpful shape bias” (Jones, 2003, p. 482).

Studying disadvantaged populations provides an important way for further research to recognize which experience results in which cognitive dispositions while taking individual differences into account. In the case of late-talking children, we think that the cognitive operation of attending to a shape is a product of a labeling frame. Children who do not develop this shape bias might need more exposure to a particular frame in order to discern their “jobs,” and thus their role (Heller and Rohlfing, in preparation). In a recent study, we showed that 3-year-old children diagnosed with language impairment benefited from repetitions of the context (Rohlfing et al., submitted). Further research should focus on children’s individual differences in applying other biases or principles in language acquisition such as the whole-object assumption (Markman and Wachtel, 1988) or mutual exclusivity (Carey and Bartlett, 1978). These differences might well be explained by children’s individual experiences with particular pragmatic frames and their individual need for a more frequent or dense occurrence of such frames.

A further question is whether pragmatic frames are relevant only for young children. We think that this is not the case. For older children and adults, Quasthoff (1997, p. 59) has applied the term “discourse unit” instead of pragmatic frame to refer to verbal behaviors performed with a specific purpose such as instructing on how to play a game, discussing, narrating a story or telling a joke in a conversation. Those behaviors exhibit a global structure that establishes sequential conditions, “not only for a single next turn but for an ordered series of next turns” (p. 58). Children and adults need to learn the global structure if they are to act appropriately (see **Table 1**).

Even though we argue that pragmatic frames are operative in later language competence (i.e., in older children as well as adults), we agree that once children possess a communicative foundation in the form of a repertoire of pragmatic frames, they can use language within them in a more elaborated way. For example, Nelson (1974, p. 277) suggests a “functional synthesis” is achieved once the child experiences various relations (pragmatic frames) in which a specific word is involved. Clearly, it is necessary to further specify the way language skills unfold within pragmatic frames (see Lieven et al., 2003, for examples) and with growing knowledge about their variety. This is not the focus here. Nonetheless, children are helped by their accumulating linguistic knowledge base, and they can also establish the association through their linguistic experience (Gershkoff-Stowe, 2002): The more words children already know, the more they can benefit from phonological and semantic memories of non-targets that spread inhibitory activation. Gershkoff-Stowe (2002, p. 665) reported that as children practice producing individual words, “those words become stronger and more resistant to interference from lexical competitors.” Thus, knowledge that is distinct to the target word minimizes word retrieval error (Capone and McGregor, 2005, p. 1469; see also Goodman et al., 1998). In line with this claim, Gershkoff-Stowe and Hahn (2007) investigated the role of practice by training 16- to 18-month-old children to comprehend and produce nouns across several sessions. Some words were trained in 12 (high-practice sets), some in 3 (medium), and some in only 1 session (low-practice sets). Compared to a control group that learned to label familiar objects, children in the experimental group not only improved their knowledge about words from high-practice sets but also demonstrated better performance in identifying low-practice words from the previous session. The authors concluded:

The more an item is selected for comprehension or production, the stronger the level of activation will be and, hence, the greater the probability of access. This idea suggests that practice with individual words in a rapidly expanding lexicon changes the operation of the lexicon through the accumulated activation of many items (p. 691).

Similarly, Thom and Sandhofer (2009) investigated the ability of 20-month-old children to extend new color words to new instances when trained with two, four, or six different color words. They found that the more broadly children learned (and the more instances they were exposed to during training), the more they were able to extend the acquired words. Thus, the “vocabulary size within a domain was related to subsequent word learning within that same domain” (Thom and Sandhofer, 2009, p. 471).

Experience that lets children bind words with their referents can lay “the ground work in infancy for more rapid (and perhaps more hypothesis-testing-like) processes in later word learning” (Smith and Yu, 2008, p. 1566). The idea here is that concepts are not isolated entities but part of semantic networks (cf. Keil and Batterman, 1984) and interactive activities. Thus, a child with extensive knowledge accretion about adjectives who is learning a new adjective for some entity is in a very different position from a child learning the same new word who has little or no knowledge of the members of that category (Keil and Batterman, 1984, p. 232; Thom and Sandhofer, 2009).

From our perspective, these examples indicate an experience not only with a particular word class but – more importantly – with its learning frame.

## Conclusion and Future Directions

We think that the concept of “pragmatic frame” helps us to understand the co-development of cognitive and communicative dispositions in children. It provides research on language acquisition with an alternative framework to the widely assumed mapping process by offering a complex context of actions and goals that enfolds at different time scales and needs to be negotiated between interactants. As outlined above, we think that our extended notion of pragmatic frames goes beyond the frameworks currently offered. Although our concept relates strongly to Bruner’s definition, we extend it by (1) clearly differentiating between the meaning and the syntax of a pragmatic frame and (2) identifying the cognitive layer. Central to our view is the assumption that pragmatic frames are not limited to the agent’s own actions, but involve pragmatic aspects of an ongoing situation organized around an interactional goal.

Can pragmatic frames be conceived as speech acts? As already noted above, pointing to an object and labeling it can be seen as a speech act, and Bruner (1983, p. 133) points to the possibility that “eventually, formats provide the basis for speech acts and their constraining felicity conditions. We learn how to invoke them by speech.” Speech act theories focus on particular linguistic constructions occurring in different pragmatic contexts. For example, the indication “it is sweet” when referring to a new candy bar can be interpreted as either a description or a warning. Which illocutionary force it has will be determined by the preceding context of the indication. Austin (1962) differentiates further between the illocutionary act (which is intended by the speaker) and the perlocutionary act (the effect of an act). In our approach, the illocutionary and perlocutionary force are present not only in the surface meaning – because this enables individuals to act and react – but also in the deep meaning – because this is formed by the underlying deep syntax. From the developmental perspective, we second Bruner (1983) and view speech acts as patterns of verbal behavior that can eventually be used explicitly. However, we are convinced that the pragmatic frame is a more basic structure underlying speech acts.

Communication practice is another concept that is less well-known in cognitive science but stems from research exploiting communication within Conversation Analysis. In linguistic research, communicative practices refer to co-constructed acts of “linguistic habitus” (Hanks, 1987, p. 668). Hanks (1987) emphasizes the cultural dependencies of such discourse practices/genres, characterizing them as “elements of linguistic habitus, consisting of stylistic, thematic, and indexical schemata on which actors improvise in the course of linguistic production” (Hanks, 1987, p. 668). This line of research focuses on the co-constructive effort involved in these practices, and as a method specifies the tasks as well as roles that individuals are fulfilling in a sequence (see, e.g., Forrester, 2013; Rossmanith et al., 2014).

Because pragmatic frames consist of a sequence of actions, they resemble the concept of scripts (Schank and Abelson, 1977). Two other related aspects are (1) the hierarchy, because scripts are described as being nested in each other (a feature we mentioned in Section “Hierarchy of Pragmatic Frames”) and (2) the presence of slots. However, to the best of our knowledge, the concept of scripts does not differentiate between the syntax and the meaning. Instead, the focus lies on the surface structure and captures the sequentiality of an event in a particular context.

To summarize the similarities and differences, we think that current approaches to semantics highlight the role of form-meaning pairings when attempting to specify which factors of the context shape the meaning. In, contrast, our approach takes a broader perspective (in time and locus) on the interaction as a source for semantics. We argue that the communicative foundation is necessary for children to learn language. We view it as emerging from (1) the routines that have become established (consisting of a particular syntax) in order to accomplish (2) a goal (i.e., the meaning) by means of (3) joint contributions split into the participants’ roles in these routines. This behavior evokes (4) cognitive dispositions. As a result, cognitive factors are embedded in the child’s social experience (Nelson, 2007, p. 45), and meaning is distributed among participants (Shotter and Newson, 1982) and among the different time scales (e.g., Rkaczaszek-Leonardi et al., 2014) encompassing memories of established routines.

For further research we propose the following hypotheses and claims:

– Individual differences in experiencing pragmatic frames (their quantity and variety) will be reflected in children’s later language skills as interactions guide them toward creating new slots or recognizing elements of frames that enable them to differentiate between known and new sequences.
– Without knowing the frame, a child’s understanding of language will be impaired.
– Learning a new frame (and thus new verbal behavior) involves a close coupling between tutor and learner. This coupling might thus be crucial for the overall success of learning.
– Children make sense of new frames by assembling known elements of the deep and surface structure.

Importantly, the concept of pragmatic frames has methodological implications. Pragmatic frames emphasize the fact that a word is not learned in a binary fashion but that the understanding of a concept and its linguistic representation emerges gradually and can be measured at different levels (Nomikou et al., 2016, accepted). As stated above, we define pragmatic frames as consisting of processes at the cognitive and pragmatic level (**Figure 1**). Accordingly, learning can be measured at those levels. More specifically,

– The development of the understanding of a new word can be measured through related cognitive functions. Visual attention within a pragmatic frame can indicate whether, for example, the goal of an action has been understood. Demonstrations of familiarity with certain objects or words indicate that a first step is being taken toward understanding the meaning of an object within an action. Motor control, such as the way in which an object is grasped, can also be an indicator of a learning process.
– The development of the understanding of communicative or pragmatic functions within a frame can be measured by expectations or behavior of the learner: for example, that feedback is expected by the tutor after demonstrating an action, and so forth.

By refocusing on the concept of pragmatic frames in this article, our main aim was to shift attention away from the learning outcome toward the learning process. Nonetheless, at the end of this article, we have to admit that some aspects remain uncovered: These pertain to the process of generalization and transfer. For example, children’s progressive use of verbal behavior needs a special focus to achieve a description of the “progressive liberation of utterances from physical contexts of co-action” (Rkaczaszek-Leonardi et al., 2015, p. 15). Empirical research has to reveal how words become powerful in enriching a situation. Whereas some work has been done on the progressively creative use of words (Barrett et al., 1991; Lieven et al., 2003), little is known about the liberation from physical contexts of co-action. Another important question is how verbal knowledge accumulated within frames becomes decontextualized and transferred from one situation to another. This focus on the development of cognitive processing can be found in some theoretical and empirical work (e.g., Nelson, 1974; Barrett et al., 1991; Rohlfing, 2006). A meta-analysis identifying word learning scenarios as particular pragmatic frames and comparing their effects might be a next helpful step toward uncovering the differences in the flexible use of verbal knowledge.

## Author Contributions

KR, BW, A-LV, and P-YO developed the framework and wrote the paper. The paper benefits from interdisciplinary collaboration and expertise in language acquistion (KR, P-YO), developmental robotics (BW, P-YO, A-LV, KR) and human–robot–interaction (A-LV, BW, P-YO).

## Conflict of Interest Statement

The authors declare that the research was conducted in the absence of any commercial or financial relationships that could be construed as a potential conflict of interest.
